# Supplementary figures and images for: DNA metabarcoding of mites from small soil samples: limited agreement with morphological identifications but improved results from long-read sequencing
Source: PeerJ. 2025 Oct 20;13:e20205. doi: 10.7717/peerj.20205 (PMC12548636; doi:10.7717/peerj.20205)

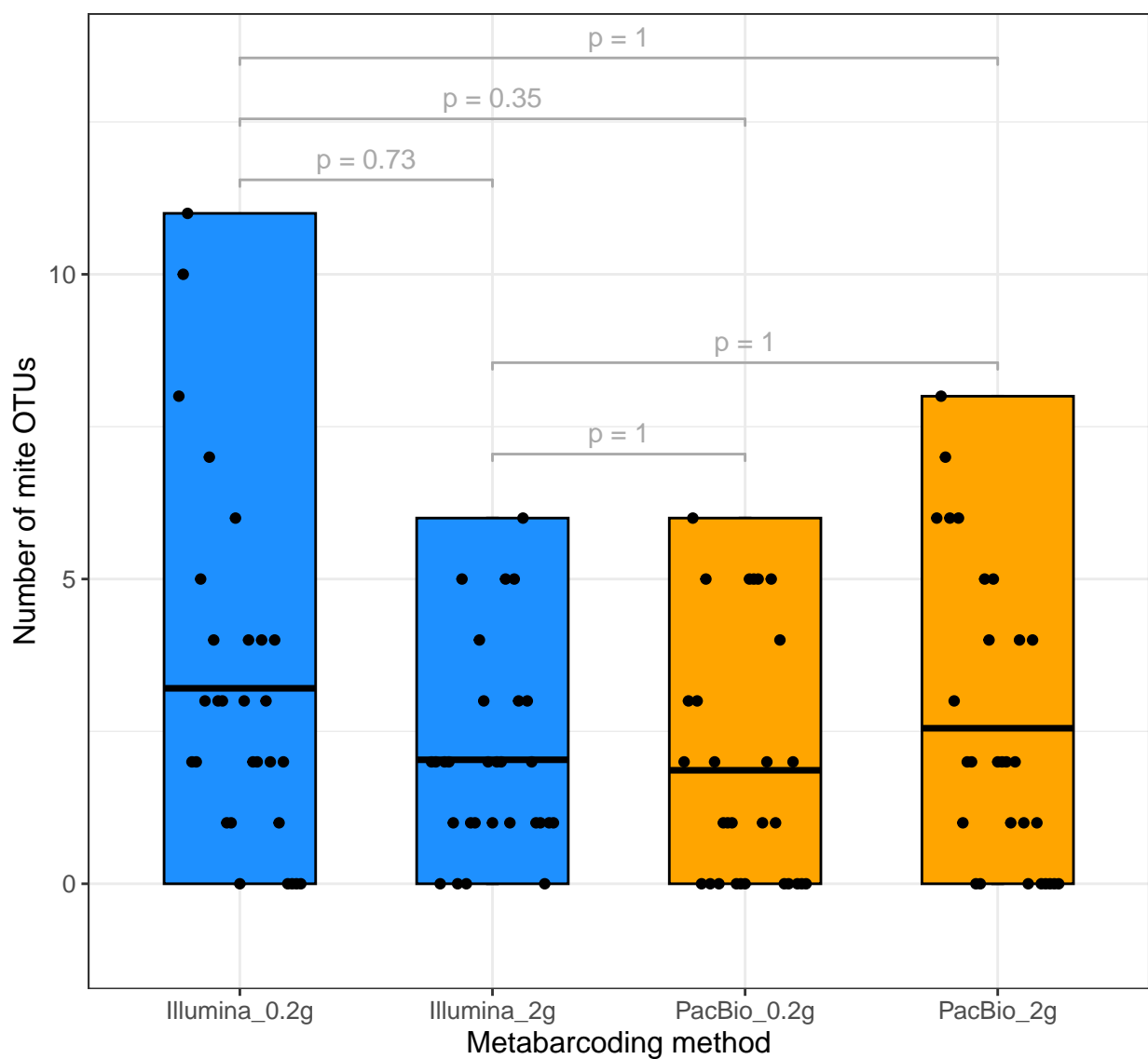

Supplement: Supplemental Information 4 [file peerj-13-20205-s004.pdf]

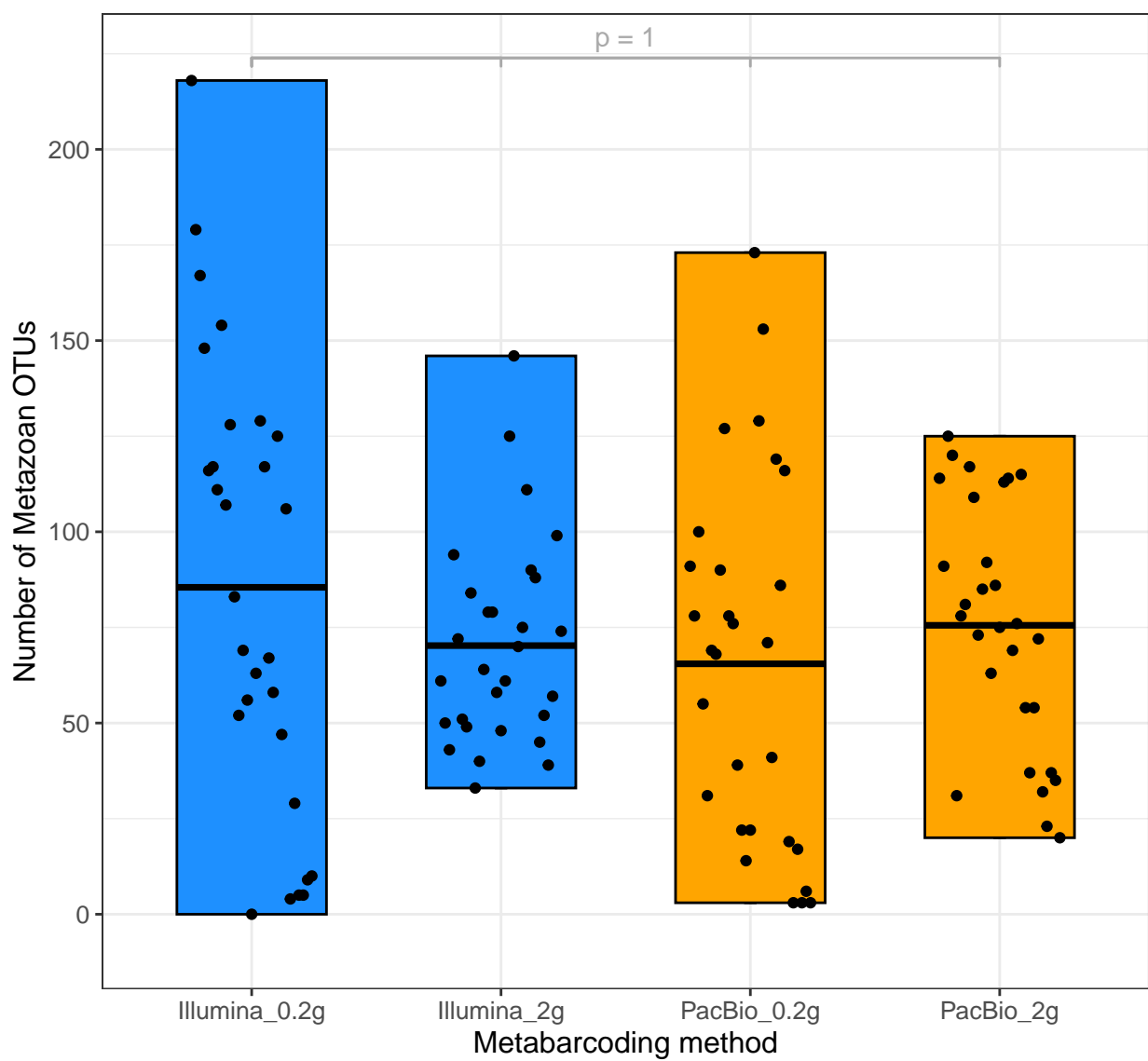

Supplement: Supplemental Information 5 [file peerj-13-20205-s005.pdf]
